# Boomerang sign in the corpus callosum and bilateral cerebellar peduncles: CLOCCs plus—a case report

**DOI:** 10.1093/bjrcr/uaaf063

**Published:** 2025-12-17

**Authors:** Gullu Tarhan, Baran Sinir, Eda Findos, Emrah Yasar

**Affiliations:** Department of Neurology, Bitlis State Hospital, Bitlis, Turkey; Department of Neurology, Tatvan State Hospital, Bitlis, Turkey; Department of Internal Medicine, Bitlis State Hospital, Bitlis, Turkey; Department of Radiology, Bitlis State Hospital, Bitlis, Turkey

**Keywords:** CLOCCs plus, corpus callosum, boomerang sign, cerebellar peduncles, cytotoxic edema

## Abstract

Cytotoxic lesions of the corpus callosum (CLOCCs) are a clinically and radiologically defined syndrome that can develop due to a wide range of etiologies, including infections, epileptic seizures, metabolic disorders, drug toxicity, malignancies, cerebrovascular diseases, and other systemic conditions. These lesions are mostly reversible but may be permanent in some cases. Although CLOCCs are typically characterized by boomerang-shaped diffusion-restricted lesions in the splenium of the corpus callosum, recent reports have shown that similar lesions can also appear in other vulnerable brain regions outside the corpus callosum and may share the same pathophysiological mechanism. Therefore, in this study, we present a rare case consistent with CLOCCs plus syndrome, showing simultaneous boomerang-shaped lesions in both the splenium of the corpus callosum and the bilateral middle cerebellar peduncles.

## Introduction

Cytotoxic lesions of the corpus callosum (CLOCCs) are clinical-radiological syndromes that were previously referred to by various names, such as reversible splenial lesion encephalopathy and reversible splenial lesion syndrome (RESLES). However, with the addition of new cases, it has been reported that the encephalopathy is not always mild and that the lesions are sometimes not reversible.^1^ In general, callosal lesions showing diffusion restriction and decreased signal on ADC are considered to result from cytotoxic edema; therefore, the term CLOCCs has been proposed as an appropriate terminology.^2^ A wide range of etiologies is responsible for the development of CLOCCs, including infections, metabolic disorders (such as hypoglycemia and hypernatremia), trauma, malignancy, medications, and cerebral venous sinus thrombosis. In a review by Starkey et al., CLOCCs were classified according to their etiologies and summarized in a table emphasizing that each presents with distinct imaging findings. In addition to splenial lesions of the corpus callosum, abnormalities in the dentate nucleus, brainstem, and thalamus may also be observed; depending on the etiology, concurrent findings such as leptomeningeal involvement, abscess, subarachnoid hemorrhage, brain contusions, and multifocal lesions have also been reported.^2^^,^^3^ Although splenial lesions are frequently reported, lesions of the middle cerebellar peduncles (MCP) are encountered more rarely. In this study, we present a case of a patient hospitalized due to pneumonia who developed and subsequently recovered from boomerang-shaped lesions simultaneously located in both the splenium of the corpus callosum and the bilateral MCP. By sharing this rare case, we aim to contribute to the existing literature.

## Case report

**Figure 1.**
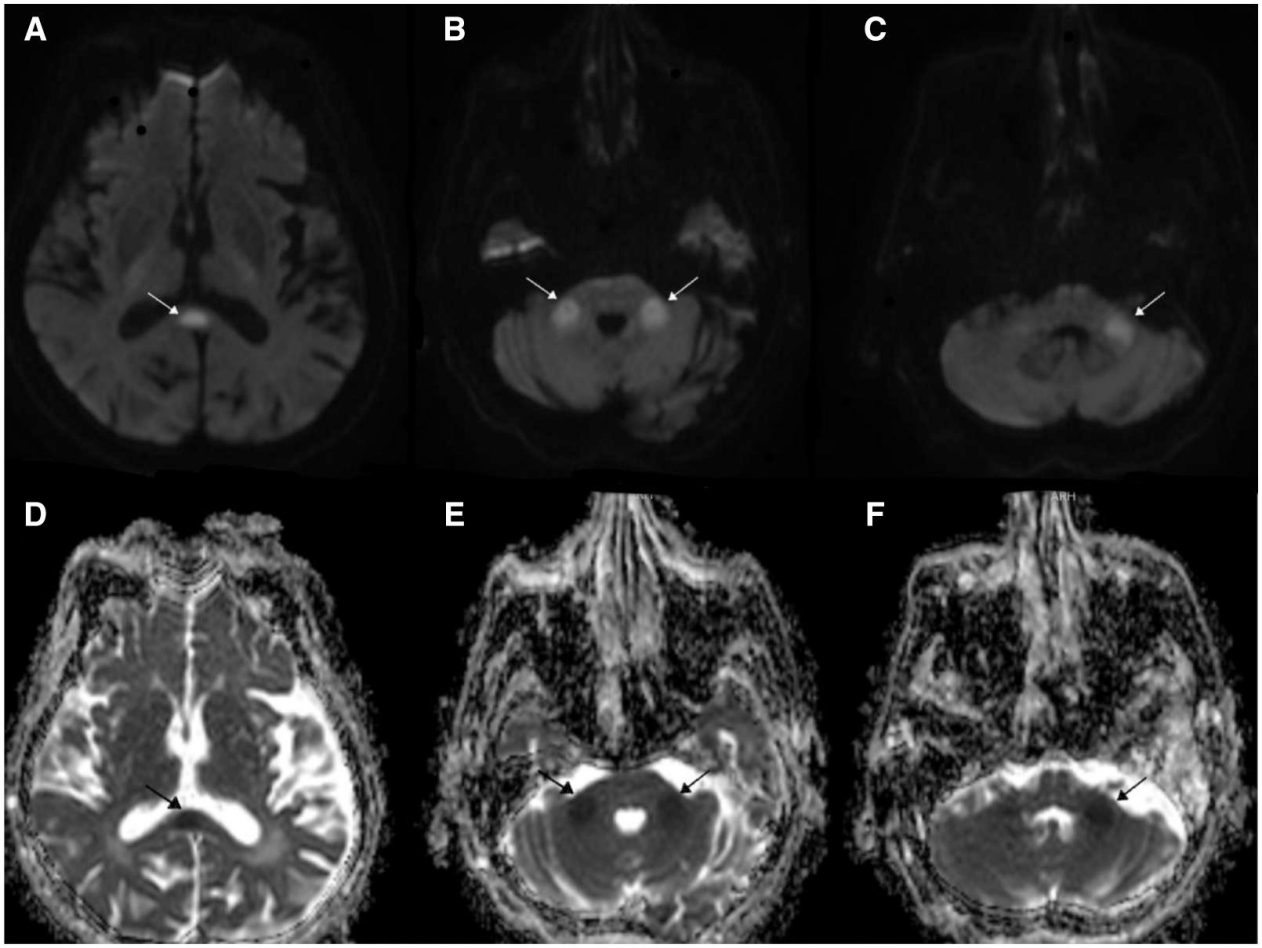
In diffusion-weighted MRI, white arrows indicate restricted diffusion: (a) shows involvement of the splenium of the corpus callosum, while (b) and (c) demonstrate similar findings in the right and left middle cerebellar peduncles (MCP), respectively. The corresponding ADC maps show hypointensity at the same locations, marked by black arrows: (d) in the splenium, and (e) and (f) in the right and left MCPs, confirming cytotoxic edema.

A 76-year-old female patient with known diagnoses of atrial fibrillation, diastolic heart failure, diabetes, hypertension, and ischemic cerebrovascular disease—who is normally able to walk with unilateral support—was brought to the emergency department due to altered consciousness and speech difficulty. Neurological exam revealed drowsiness with no eye-opening to stimuli; passive eyelid opening showed midline gaze with preserved visual tracking, consistent with eye-opening apraxia. Pupils were isochoric with positive light reflexes. The left arm moved spontaneously, while the right arm and both legs withdrew due to pain. Right-sided spasticity was noted from a prior stroke, reflexes were absent, and Babinski responses were indifferent bilaterally. Laboratory findings revealed elevated acute-phase reactants, increased sputum secretion, and low-grade fever. Although oxygen saturation was decreased, other vital signs were stable. Thoracic CT showed bilateral pleural effusion and ground-glass opacities. Diffusion-weighted MRI demonstrated diffusion restriction in the splenium of the corpus callosum and bilateral MCP, consistent with cytotoxic edema as seen on the ADC map. The affected areas appeared hyperintense on FLAIR and iso- to mildly hypointense on T1-weighted images, without associated mass effect or hemorrhage. These features are compatible with transient cytotoxic lesions, typically observed in reversible callosal and cerebellar involvement (Figure 1).

**Figure 2.**
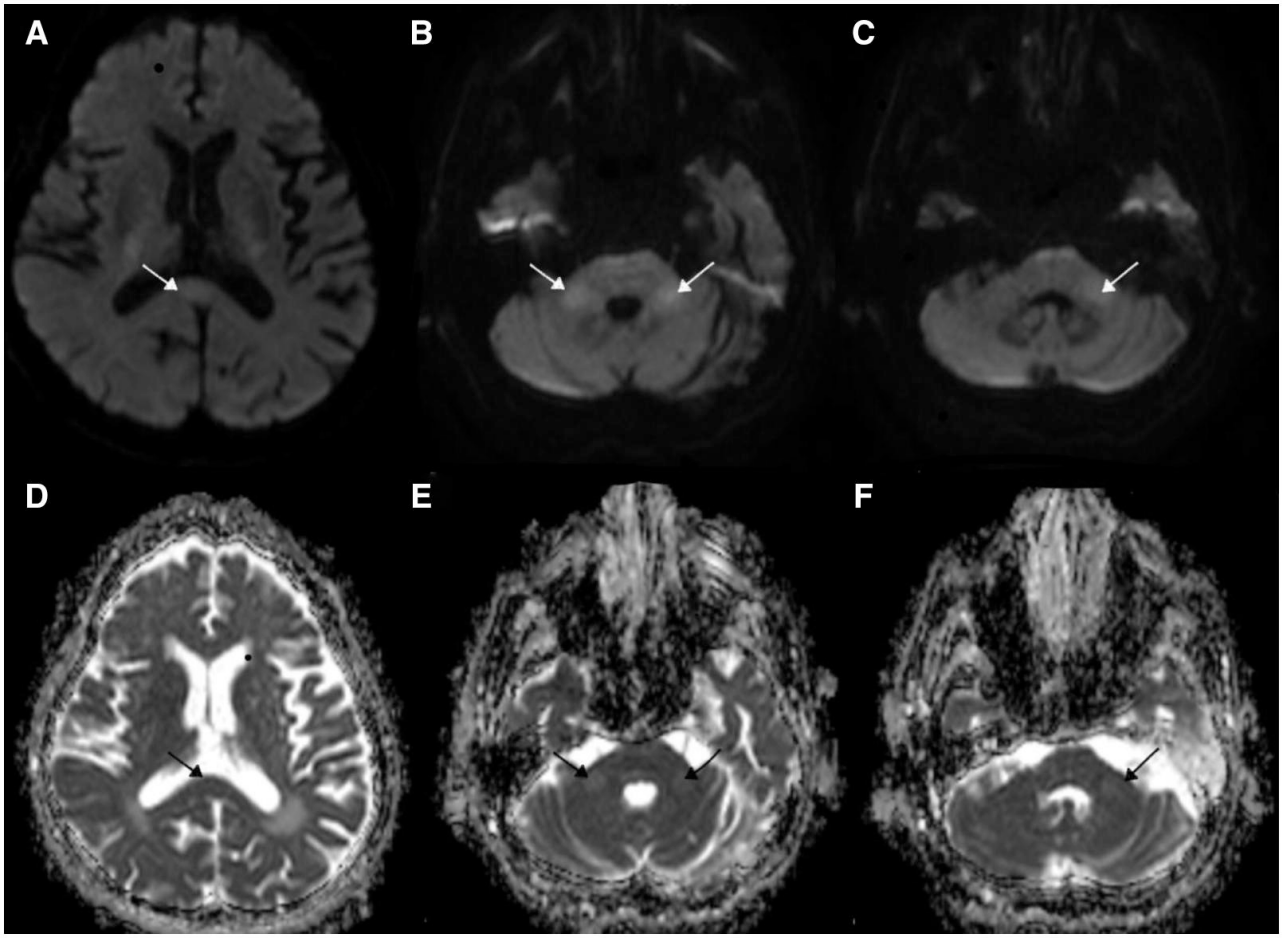
Images a–c show near-complete resolution of previous diffusion restriction in the splenium of the corpus callosum (a) and bilateral middle cerebellar peduncles (MCP) (b and c), as indicated by white arrows. Corresponding ADC maps d–f demonstrate normalization of signal intensity in the same regions (black arrows), consistent with near-total resorption of cytotoxic edema.

The patient was admitted to the intensive care unit, and blood, urine, and sputum cultures were obtained. Empirical antibiotic therapy was initiated. EEG performed due to semicoma showed 4.5-7 Hz theta waves, with occasional generalized spikes, polyspikes, and triphasic waves accompanying the background activity. These findings were interpreted as supportive of encephalopathy, and Levetiracetam 500 mg twice daily was started. Although cultures were negative, clinical improvement and reduced inflammatory markers were noted. She returned to baseline neurological status and was discharged from the ICU. A follow-up diffusion MRI obtained 3 weeks later demonstrated near-complete resolution of the previously observed diffusion-restricted lesions in the splenium and bilateral MCP, accompanied by normalization of ADC values and substantial decrease in FLAIR signal intensity, consistent with the transient nature of cytotoxic lesions (Figure 2).

The encephalopathic picture was considered secondary to an underlying infection, with imaging showing cytotoxic edema consistent with CLOCCs and similar lesions in the MCP. Despite initial improvement, the patient’s condition worsened, though a repeat MRI showed no new findings. MRSA was later detected in blood cultures, prompting broad-spectrum antibiotics. Worsening renal function led to ICU readmission, and after 2 months of intensive care, the patient suffered a cardiac arrest and was declared exitus despite resuscitation efforts.

## Discussion

CLOCCs is an imaging finding that usually resolves within a month and can result from various conditions such as infections, seizures, metabolic disorders, drug toxicity, malignancies, and cerebrovascular diseases.^4^ Our findings are in line with the recent systematic review by Moors et al., which provided a comprehensive overview of the diverse etiologies and imaging characteristics of CLOCCs, further supporting our observations.^5^ The proposed mechanism underlying this condition involves the excitotoxic effects of glutamate on various receptors and ion pumps, leading to the influx of water into astrocytes and neurons, which results in intracellular edema and diffusion restriction.^6^ The corpus callosum, particularly the splenium, is more susceptible to cytokineopathy and toxic effects due to its high receptor density.^7^ MCP is a region rich in metabolically active axons, through which the cortico-ponto-cerebellar pathways pass between the pons and cerebellar hemispheres, making it one of the structures most vulnerable to damage in cases of hypoxia, toxin exposure, or metabolic stress.^8^ The presence of ground-glass haziness in the chest suggests a possible viral etiology, whereas MRSA infection may have been iatrogenic due to prolonged hospitalization. Although a direct infectious agent was not identified in our patient, pneumonia was diagnosed based on imaging and laboratory findings. The reason for the development of brain involvement in this case, and whether it is related to the severity of the infection, remains open to discussion. Furthermore, despite the presence of multiple pre-existing comorbidities in our patient, similar lesions have also been reported in the literature in young individuals without any underlying medical conditions.^9^ Although treatment of this condition has generally been recommended in the literature as addressing the underlying cause, improvement with IVIG and methylprednisolone has been reported in a case associated with a specific agent, such as COVID-19.^10^ As seen in our patient, it is unclear whether treating the underlying cause alone is sufficient, and the role of immunomodulatory therapies in recovery remains debated. This is particularly important in cases where the exact etiology remains uncertain or a direct infectious agent cannot be identified, making the role and effectiveness of such supportive treatments critical in the clinical decision-making process. Therefore, there is a need for more comprehensive studies to evaluate the potential benefits of supportive therapies in patients presenting with similar neuroimaging findings.

## Conclusion

With this case report, we propose the term “CLOCCs plus syndromes” to describe cases in which lesions referred to as CLOCCs also involve other brain regions through similar mechanisms. Additionally, treating the underlying cause of these lesions may not always be sufficient, and the effectiveness of supportive immunomodulatory therapies remains a subject that requires further investigation.

## Learning Points

- CLOCCs may extend beyond the splenium, involving additional callosal or cerebellar regions we therefore propose the term “CLOCCs-plus syndrome.”
- Radiological reversibility does not necessarily indicate complete clinical recovery, highlighting the need for long-term follow-up.
- Persistent lesions despite treatment suggest that structural changes can outlast metabolic resolution.
- Immunomodulatory therapy may be beneficial in selected cases with prolonged inflammatory activity.

## Data Availability

All relevant data supporting the findings of this case report are included within the article.
